# Prevalence and Characteristics of Isolated Nighttime Masked Uncontrolled Hypertension in Treated Patients

**DOI:** 10.3390/medicina60091522

**Published:** 2024-09-18

**Authors:** Kang Hee Kim, Jaehoon Chung, Suyoung Jang, Byong-Kyu Kim, Masanori Munakata, Moo-Yong Rhee

**Affiliations:** 1 Department of Cardiology, Dongguk University Ilsan Hospital, Goyang-si 10326, Gyeonggi-do, Republic of Korea; csmkkh@hotmail.com (K.H.K.); jhchung@dumc.or.kr (J.C.); catherine9@hanmail.net (S.J.); 2 College of Medicine, Dongguk University, Gyeongju-si 38066, Gyeongsangbuk-do, Republic of Korea; bleumatin@gmail.com; 3 Division of Hypertension and Research Center for Lifestyle-Related Disease, Tohoku Rosai Hospital, Sendai 981-8563, Japan; munakata@tohokuh.johas.go.jp

**Keywords:** masked hypertension, nighttime, diastolic, ambulatory blood pressure

## Abstract

*Background and Objectives*: We evaluated the prevalence and characteristics of isolated nighttime masked uncontrolled hypertension (IN-MUCH) in treated patients. *Materials and Methods*: Participants aged 20 years or older who were on antihypertensive medication underwent three-day office blood pressure (BP) and 24 h ambulatory BP measurements. Hypertension phenotypes were classified as controlled hypertension (CH), isolated daytime masked uncontrolled hypertension (ID-MUCH), IN-MUCH, and daytime and nighttime masked uncontrolled hypertension (DN-MUCH). *Results*: Among 701 participants, 544 had valid BP data and controlled office BP (<140/90 mmHg). The prevalence of IN-MUCH was 34.9%, with a higher prevalence of men and drinkers than in those with CH. Patients with IN-MUCH had higher office systolic BP (SBP) and diastolic BP (DBP) than those with CH. The prevalence of IN-MUCH was 37.6%, 38.5%, and 27.9% in patients with optimal, normal, and high-normal office BP levels, respectively. Among IN-MUCH patients, 51.6% exhibited isolated uncontrolled DBP and 41.1% uncontrolled SBP and DBP. Younger age (*p* = 0.043), male sex (*p* = 0.033), and alcohol consumption (*p* = 0.011) were more prevalent in patients with isolated uncontrolled DBP than in those with uncontrolled SBP and DBP. Age and alcohol consumption were positively associated, whereas high-normal office BP exhibited a negative association with IN-MUCH. *Conclusions*: The IN-MUCH was significantly more prevalent in patients with normal or optimal office BP, posing treatment challenges. Further investigation is needed to determine whether differentiation between isolated uncontrolled DBP and combined uncontrolled SBP and DBP is necessary for prognostic assessment of IN-MUCH.

## 1. Introduction

Hypertension is a well-known risk factor for cardiovascular morbidity and mortality. The advent of ambulatory blood pressure (BP) measurements has led to accumulating evidence demonstrating its superior cardiovascular-predictive power [1,2]. Furthermore, ambulatory BP measurements have facilitated the identification of various hypertension phenotypes, including white-coat, masked, and sustained hypertension [3].

Ambulatory BP measurement offers the advantage of BP monitoring during sleep. An additional subtype of masked hypertension, identified through nighttime BP measurement, is isolated nighttime hypertension (INH), characterized by elevated nighttime BP and normal daytime levels [4,5]. Diagnosing isolated nighttime hypertension requires ambulatory or home BP measurement. However, the routine use of ambulatory or home BP measurement devices capable of measuring BP during sleep poses challenges in clinical practice. The reported prevalence of isolated nighttime hypertension has ranged from 10% to 20% [5,6,7].

Studies have emphasized the association between isolated nighttime hypertension and elevated risks of total mortality, cardiovascular events, and renal complications [8,9]. We reported the characteristics of INH in the untreated population [5]. However, the characteristics of isolated nighttime masked uncontrolled hypertension (IN-MUCH) in treated patients remain uncertain. Identifying characteristics suggestive of IN-MUCH during hypertension treatment in clinical practice would be beneficial.

Recent analyses of isolated diastolic hypertension (IDH) suggest that it influences the occurrence of cardiovascular events, indicating that it may not be an innocent condition [10,11]. Masked hypertension can be further classified based on systolic and diastolic blood pressure. Untreated patients with systolic and systolic/diastolic masked hypertension were found to have significantly higher carotid intima-media thickness compared to those with isolated diastolic masked hypertension [12]. However, it remains unclear whether the prognosis of systolic and systolic/diastolic masked hypertension differs from that of isolated diastolic masked hypertension based solely on carotid intima-media thickness. Therefore, considering that patients with masked hypertension form a heterogeneous group, further research is required to determine whether they confer the same degree of cardiovascular risk.

To date, the characteristics of IN-MUCH have not been thoroughly investigated. No studies have investigated the extent to which isolated uncontrolled diastolic BP (DBP) influences the prevalence and characteristics of masked hypertension. This study aimed to identify the prevalence and characteristics of patients with IN-MUCH. Additionally, we assessed the prevalence and characteristics of IN-MUCH with uncontrolled DBP, as well as isolated daytime (ID-MUCH) and day–night masked uncontrolled hypertension (DN-MUCH).

## 2. Materials and Methods

### 2.1. Study Subjects

This was a cross-sectional, community-based study. The primary objectives and study protocol are described in detail elsewhere [13]. Between August 2018 and April 2020, 701 participants were recruited from Goyang-si and Paju-si, Gyeonggi Province, South Korea. Recruitment was conducted through postcards distributed to households and posters displayed at hospitals, public health centers, and social facilities.

We recruited individuals aged ≥20 years on antihypertensive medication for more than one year. The exclusion criteria included secondary hypertension, hypertensive emergency disease, severe heart failure (NYHA class III-IV), a recent diagnosis of cardiovascular disease within six months, clinically significant arrhythmias (such as ventricular tachycardia, atrial fibrillation, atrial flutter), and any conditions deemed by the investigator to interfere with study completion.

Among the participants, patients with controlled hypertension by office BP were analyzed in this study.

### 2.2. Study Protocol

On the first visit, the participants underwent simultaneous office BP measurements for each arm at the clinical trial center. They also completed a questionnaire detailing their clinical and demographic characteristics, including self-reported history of cardiovascular disease (CVD), diabetes, alcohol consumption, smoking status, the duration of hypertension treatment, and antihypertensive medication. After completing the questionnaire, the participants wore an ambulatory BP monitor for 24–25 h. The next day, they returned to the center to remove the monitor and measure office BP. Additional office blood pressure measurements were obtained during a subsequent visit a few days later.

After at least eight hours of overnight fasting, various parameters were measured, including fasting blood glucose, HbA1c, lipid profile, renal function markers, and urine microalbumin levels. The estimated glomerular filtration rate (eGFR, mL/min/1.73 m^2^) was calculated using the 2009 Chronic Kidney Disease-Epidemiology Collaboration (CKD-EPI) creatinine equation [14]. A 12-lead resting electrocardiography was also performed. Total daily alcohol consumption (g/day) was calculated as follows: drinking days per week × number of glasses in one sitting × 7 g (amount of alcohol contained in one standard glass). Participants were divided into none, low-, and high-consumption groups based on the median value of total daily alcohol consumption (6.53 g/day).

Written informed consent was obtained from all the participants before their enrollment in the study. The study protocols and informed consent forms were reviewed and approved by the Institutional Review Board of Dongguk University Ilsan Hospital (DUIH 2018-02-013-002). This study was registered at ClinicalTrials.gov (registration no: NCT03868384).

### 2.3. Measurement of Office and Ambulatory BP

At each visit, trained nurses measured office BP in a quiet room according to the standard method, using a validated oscillometric device (WatchBP Office; Microlife, Taipei, Taiwan) to measure BP in both arms simultaneously. BP was measured three times at one-minute intervals following five minutes of seated rest with an appropriately sized cuff. The participants were instructed to avoid smoking and caffeine and exercise 30 min before the measurements. The average of nine readings (three at each visit) for each arm was calculated, with the arm showing the higher average BP considered the index arm.

Ambulatory blood pressure monitoring over 24 to 25 h was conducted on the non-dominant arm using an automated, noninvasive oscillometric device (Mobil-O-Graph, I.E.M GmbH, Stolberg, Germany) with a measurement interval of 30 min. Participants were instructed to maintain normal daily activities. Daytime was defined as the period from 09:00 to 21:00 h and nighttime from 00:00 to 06:00 h. Valid measurements were defined as successful readings for more than 70% of the total measurement attempts, including at least 14 measurements during the daytime period and at least seven measurements during the nighttime period. Average values of 24 h, daytime, and nighttime BP were calculated.

### 2.4. Definition of Office and Ambulatory BP Control Status

Controlled hypertension by office BP, controlled hypertension (CH), masked uncontrolled hypertension (MUCH), ID-MUCH, IN-MUCH, DN-MUCH, isolated uncontrolled systolic blood pressure (SBP), isolated uncontrolled DBP, and uncontrolled SBP and DBP are defined in Table 1. Office BP levels were categorized as follows. “Optimal” office BP level was defined as office SBP < 120 mmHg and office DBP < 80 mmHg; “normal” office BP level was defined as 130 mmHg > office SBP ≥ 120 mmHg and/or 85 mmHg > office DBP ≥ 80 mmHg; “high normal” office BP level was defined as 140 mmHg > office SBP ≥ 130 mmHg and/or 90 mmHg > office DBP ≥ 85 mmHg.

### 2.5. Statistical Analysis

All data are presented as numbers (percentages) or mean ± standard deviation. Comparisons of baseline clinical and demographic characteristics between the groups were performed using the unpaired *t*-test comparison of the two groups (continuous variables) and the Chi-square test (categorical variables) with post hoc pairwise comparison at the Bonferroni-corrected alpha level of 0.0125 or the Kruskal–Wallis test with the Bonferroni-corrected multiple tests.

When comparing 24 h SBP and DBP variability expressed as standard deviation (SD) between patients with isolated uncontrolled DBP and uncontrolled SBP and DBP among those with IN-MUCH, age and sex were adjusted as covariates with Bonferroni correction. The relative risks for IN-MUCH were estimated based on clinical and demographic characteristics. Age, sex, body mass index, smoking status, alcohol consumption, history of CVD, diabetes mellitus, office BP level categories, and eGFR were included using a forward stepwise method.

For statistical analyses, MedCalc software version 22.020 (MedCalc Software Ltd., Ostend, Belgium) and SigmaPlot 13.0 (Grafiti LCC, Palo Alto, CA, USA) were used.

## 3. Results

### 3.1. Patients Characteristics

Of the 701 participants recruited, we analyzed data from 544 patients with valid ambulatory and office BP data and controlled office BP. Specifically, among these 544 participants, 31.8% (*n* = 173) had CH, and 68.2% (*n* = 371) had MUCH.

Table 2 outlines the demographic and clinical characteristics of the patients. Within the study population, 31.8% (*n* = 173) had CH, 3.3% (*n* = 18) had ID-MUCH, 34.9% (*n* = 190) had IN-MUCH, and 30.0% (*n* = 163) had DN-MUCH. Patients with MUCH were younger than those with CH (*p* = 0.018). The prevalence of males and drinkers was higher among patients with MUCH than those with CH (*p* < 0.001 for both). Patients with IN-MUCH consistently showed a higher prevalence of males and drinkers compared to those with CH, along with higher alcohol consumption.

A more significant percentage of patients with MUCH were taking calcium channel blockers and beta-blockers (68.2% vs. 54.3%, *p* = 0.002, 14.3% vs. 7.5%), while fewer were taking angiotensin receptor blockers (69.8% vs. 80.9%, *p* = 0.006) than those with CH. No differences were observed in the classes of antihypertensive medications between the CH and IN-MUCH patients (Table 3).

Office SBP and DBP were higher in patients with MUCH than those with CH (*p* < 0.001 for both). Patients with IN-MUCH and DN-MUCH exhibited higher office SBP and DBP than patients with CH (Table 4).

### 3.2. Prevalence of Isolated Nighttime Masked Uncontrolled Hypertension

**Figure 1:**
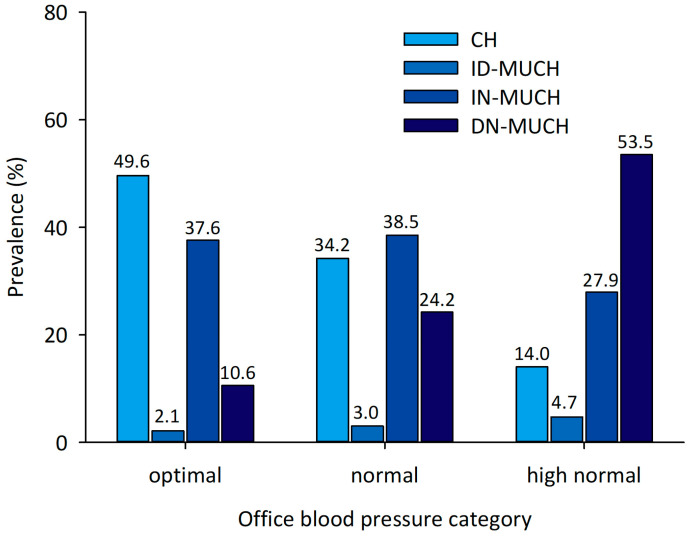
Prevalence of hypertension phenotypes according to office blood pressure categories. CH, controlled hypertension; ID-MUCH, isolated daytime masked uncontrolled hypertension; IN-MUCH, isolated nighttime masked uncontrolled hypertension; DN-MUCH, daytime–nighttime masked uncontrolled hypertension.

The prevalence of IN-MUCH was 37.6%, 38.5%, and 27.9% in patients with optimal, normal, and high normal office BP, respectively (Figure 1). On the contrary, the prevalence of DN-MUCH was the highest in patients with high normal office BP.

**Figure 2:**
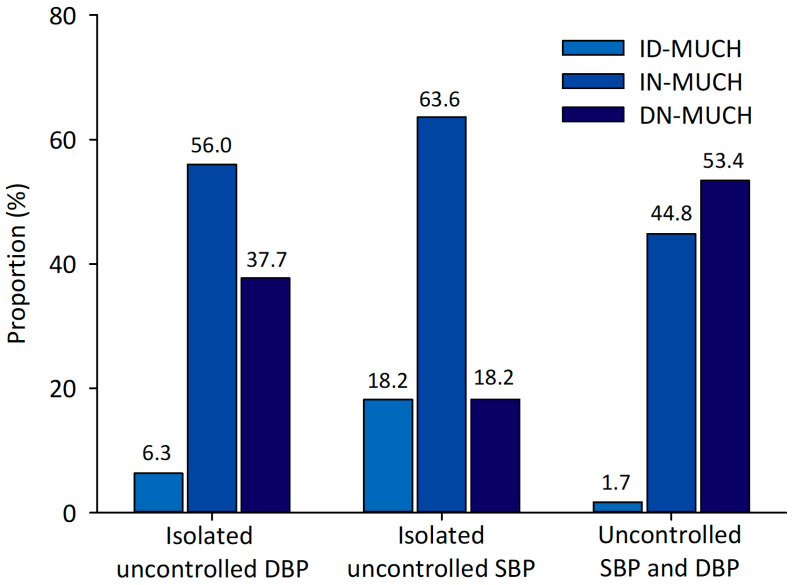
Distribution of masked uncontrolled hypertension phenotypes according to the controlled status of systolic and diastolic blood pressure. ID-MUCH, isolated daytime masked uncontrolled hypertension; IN-MUCH, isolated nighttime masked uncontrolled hypertension; DN-MUCH, daytime–nighttime masked uncontrolled hypertension; SBP, systolic blood pressure; DBP, diastolic blood pressure.

Among patients with isolated uncontrolled DBP, constituting 32.2% of the study population (*n* = 175), 56.0%, 6.3%, and 37.7% exhibited IN-MUCH, ID-MUCH, and DN-MUCH, respectively (Figure 2).

### 3.3. Isolated Nighttime Masked Uncontrolled Hypertension and Isolated Uncontrolled Diastolic Blood Pressure

**Figure 3:**
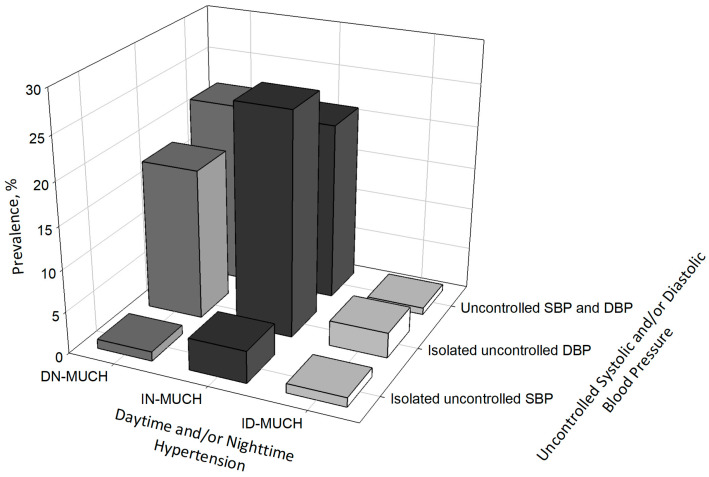
Distribution of isolated daytime/nighttime masked uncontrolled hypertension and isolated uncontrolled systolic/diastolic blood pressure. ID-MUCH, isolated daytime masked uncontrolled hypertension; IN-MUCH, isolated nighttime masked uncontrolled hypertension; DN-MUCH, daytime–nighttime masked uncontrolled hypertension; SBP, systolic blood pressure; DBP, diastolic blood pressure.

Among patients with MUCH, the prevalence of isolated uncontrolled DBP and uncontrolled SBP and DBP was 26.4% vs. 21.0% in patients with IN-MUCH and 17.8% vs. 25.1% in patients with masked DN-MUCH (Figure 3).

Within the patients with IN-MUCH, 51.6% had isolated uncontrolled nighttime DBP, 7.4% had isolated uncontrolled nighttime SBP, and 41.1% had uncontrolled nighttime SBP and DBP. The clinical and demographic characteristics of patients with isolated uncontrolled nighttime DBP and uncontrolled nighttime SBP and DBP were compared among IN-MUCH patients (Table 5). Patients with isolated uncontrolled nighttime DBP exhibited a lower mean age (*p* = 0.043) and higher prevalence of males (*p* = 0.033) and drinkers (*p* = 0.011) than those with uncontrolled nighttime SBP and DBP. There was no difference in the prevalence of CVD, diabetes mellitus, and chronic kidney disease (eGFR < 60 mL/min/1.73 m^2^) between these groups. Notably, the SD of 24 h SBP was higher in patients with uncontrolled nighttime SBP and DBP than in those with isolated uncontrolled nighttime DBP, the difference that persisted even after adjusting for age and sex (*p* = 0.003).

### 3.4. Determinants of Isolated Nighttime Masked Uncontrolled Hypertension

The multivariate logistic regression analysis revealed a positive association of age (OR: 1.04, 95%CI: 1.02 to 1.07) and alcohol consumption (OR: 1.60, 95%CI: 1.10 to 2.35) with IN-MUCH, while high normal office BP (OR: 0.59, 95%CI: 0.39 to 0.88) and smoking (OR: 0.46, 95%CI: 0.21 to 0.10) showed a negative association. Isolated uncontrolled DBP (OR: 37.96, 95%CI: 18.76 to 76.830) and uncontrolled SBP and DBP (OR: 20.71, 95%CI: 10.38 to 41.32) were significantly associated with IN-MUCH. The use of angiotensin-converting enzyme inhibitors, angiotensin receptor blockers, beta-blockers, calcium channel blockers, and diuretics was not associated with IN-MUCH.

## 4. Discussion

The INH, characterized by normotension during the day and elevated BP at night, poses a heightened risk of cardiovascular events and overall mortality compared to nighttime normotension [8]. However, the identification of INH is challenging. Compared to our previous study of INH in the untreated general population, IN-MUCH in treated patients (34.9%) seemed more prevalent than INH in the untreated general population (22.8%) [5]. The prevalence of IN-MUCH in treated patients with an optimal range of office BP was higher (37.6%) than that of INH in untreated patients with controlled office BP (17%). The discrepancy may be attributed to the common practice among many treated patients taking antihypertensive medication in the morning. While morning administration of antihypertensives seems to control office BP, nighttime BP may remain uncontrolled [15].

DN-MUCH showed the highest prevalence in patients with high normal office BP. This can also be explained by the administration of insufficient doses of antihypertensive drugs in the morning. Office BP was measured in the morning without restricting the intake of antihypertensive medications. As Franklin et al. discussed [16], antihypertensive medications are typically taken in the morning. Measurement of office BP in the morning may reflect the peak level of antihypertensive drugs, appearing normal. However, BP may rise as the medication concentration decreases to the trough levels in the late daytime or nighttime. Therefore, if office BP exceeds 130/80 mmHg, the dose of antihypertensive drugs is insufficient, leading to poor control of late daytime blood pressure and possibly resulting in a higher frequency of DN-MUCH. On the other hand, if the morning office BP is below 130/80 mmHg, the level of medication may remain adequate during the day to maintain BP control. However, BP may rise as the drug level decreases at night, potentially explaining the high prevalence of IN-MUCH [15].

The high prevalence of IN-MUCH in patients with a normal and optimal range of office BP presents a significant challenge in hypertension management, as assessing nighttime BP through 24 h ambulatory monitoring or specialized home BP devices capable of measuring nighttime BP is difficult. Consequently, identifying distinctive characteristics associated with a high probability of IN-MUCH could greatly enhance clinical practice. In this study, patients with IN-MUCH exhibited a higher prevalence of male sex and alcohol consumption compared to those with CH. Various observational studies and meta-analyses have shown a link between alcohol consumption and higher prevalence and incidence of hypertension [17,18]. Despite controversies regarding the amount of alcohol consumed [19], a recent meta-analysis reported a linear association between the amount of alcohol consumed and SBP [20]. Several studies have highlighted the time-dependent biphasic effect of acute alcohol consumption on BP, with a decrease in the first hours and an increase after 12 h of intake [21,22]. Furthermore, habitual alcohol intake has been associated with increased morning BP while leaving nighttime BP relatively unaffected [23,24]. However, this study diverges from prior studies, showing a higher prevalence and amount of alcohol consumption in patients with IN-MUCH compared to those with CH. Notably, patients were advised to abstain from alcohol during their 24 h ambulatory BP monitoring, minimizing the acute effect of alcohol on BP. Given the higher representation of men in the IN-MUCH group and the more significant proportion of male drinkers compared to women (71.9% vs. 23.9%, *p* < 0.001), the potential influence of sex on IN-MUCH cannot be discounted.

The substantial contribution of isolated uncontrolled DBP to the IN-MUCH, as well as the difference in 24 h SBP SD between patients with isolated uncontrolled DBP and those with combined uncontrolled SBP and DBP, were noteworthy findings. More than half of the patients with IN-MUCH had isolated uncontrolled DBP. Despite previous research indicating an association between isolated diastolic hypertension determined by standardized office BP and CVD risk [25,26,27], the effect was small to modest compared to combined systolic–diastolic hypertension. Notably, we diagnosed hypertension based on ambulatory BP. The Analysis of the International Database on Ambulatory Blood Pressure in Relation to Cardiovascular Outcomes (IDACO) database has only evaluated the prognostic significance of isolated diastolic hypertension defined using ambulatory BP [10,11]. In the analysis of the IDACO database, isolated diastolic hypertension defined using the 2018 European Society of Cardiology/European Society of Hypertension guidelines definition of ambulatory BP diagnostic threshold was associated with an increased risk of cardiovascular events across all age groups [10,11]. However, when hypertension was defined based on nighttime BP, isolated systolic hypertension and combined systolic and diastolic hypertension were significantly associated with total mortality and cardiovascular (CV) events, whereas isolated diastolic hypertension did not exhibit such association [10]. As indicated by the IDACO data analysis and the difference in 24 h SBP SD in our study, the prognostic implication of IN-MUCH by isolated uncontrolled DBP seems to have the potential to differentiate from IN-MUCH with uncontrolled SBP and DBP. Therefore, prospective outcome-based studies or cohort data analyses should be pursued to elucidate the prognostic significance of IN-MUCH specifically associated with isolated uncontrolled DBP.

Strategies such as chronotherapy and moderation of alcohol intake could be considered to address the prevalence of IN-MUCH among patients. While many patients adhere to morning antihypertensive drug regimens, effectively controlling their office BP and daytime ambulatory BP, there remains a possibility wherein nighttime BP levels remain uncontrolled [28]. Consequently, the potential application of chronotherapy in reducing IN-MUCH can be considered a plausible approach. However, its efficacy poses challenges as debate persists regarding the effectiveness of morning versus bedtime dosing in reducing nighttime BP and CVD with chronotherapy [29,30]. Although the potential influence of sex on IN-MUCH warrants consideration, the notable correlation of alcohol consumption with IN-MUCH emphasizes the need for further research on the effect of aggressive moderation or complete abstinence from alcohol in treating IN-MUCH.

Our study has several limitations and strengths that should be mentioned. First, the primary purpose of this study was to evaluate the effectiveness of home BP measurement on BP control. As such, the study was not designed to identify the characteristics of patients with IN-MUCH. Moreover, the study participants were not recruited using a representative population sampling method. The sample size may be insufficient, as it was not specifically calculated for this study. Consequently, extrapolating the findings of this study necessitates further investigations in diverse and larger population cohorts to facilitate a more comprehensive generalization of the results. Second, this cross-sectional study could not evaluate the outcome value of isolated uncontrolled nighttime DBP. Therefore, prospective outcomes or cohort studies should evaluate the prognostic significance of IN-MUCH determined by isolated uncontrolled nighttime DBP. Third, IN-MUCH was determined based on a single measurement of 24 h ambulatory BP. Concerns regarding the short-term reproducibility of nighttime hypertension have been raised: very good (κ = 0.85) among treated individuals but poor concordance (κ = 0.21) among untreated individuals [31,32]. The number of 24 h ambulatory BP measurements required to establish reproducibility has not yet been evaluated. Moreover, the prognostic difference between individuals who initially presented with IN-MUCH at the first 24 h ambulatory BP measurement but changed to CH at subsequent measurements or vice versa and those with persistent CH or IN-MUCH throughout the measurement sessions has not been investigated. However, the significant association between INH and CVD outcomes has been substantiated based on a single measurement of 24 h ambulatory BP [8,9].

The significance of this study lies in proposing new hypertension phenotypes, i.e., a combination of isolated diurnal and isolated systolic or diastolic hypertension, in addition to the existing phenotypes of masked hypertension and nighttime hypertension (Figure 3). The significant contribution of isolated uncontrolled DBP among those with IN-MUCH is a noteworthy finding. As mentioned above, the clinical implications of this new combination of hypertension phenotypes should be investigated in prospective outcome-based studies or cohort data analyses.

## 5. Conclusions

In conclusion, the considerable occurrence of IN-MUCH in patients with normal and optimal office BP poses treatment challenges. While the prognostic significance of IN-MUCH with isolated uncontrolled DBP was not determined, further investigation is needed to determine whether differentiation between isolated uncontrolled DBP and combined uncontrolled SBP and DBP is necessary for the prognostic assessment of IN-MUCH.

## Figures and Tables

**Table 1:** Definition of hypertension phenotypes.

Controlled Hypertension by Office BP
Office BP < 140/90 mmHg
Office BP level
Optimal: SBP < 120 and DBP < 80 mmHg
Normal: SBP 120–129 and/or DBP 80–84 mmHg
High normal: SBP 130–139 and/or DBP 85–89 mmHg
Controlled hypertension (CH)
Office BP < 140/90 mmHg and
24 h averaged BP < 130/80 mmHg and
Daytime averaged BP < 135/85 mmHg and
Nighttime averaged BP < 120/70 mmHg
Masked uncontrolled hypertension (MUCH)
Office BP < 140/90 mmHg and
24 h averaged BP ≥ 130/80 mmHg and/or
Daytime averaged BP ≥ 135/85 mmHg and/or
Nighttime averaged BP ≥ 120/70 mmHg
Isolated daytime masked uncontrolled hypertension (ID-MUCH)
Office BP < 140/90 mmHg and
Daytime averaged BP ≥ 135/85 mmHg and
Nighttime averaged BP < 120/70 mmHg
Isolated nighttime masked uncontrolled hypertension (IN-MUCH)
Office BP < 140/90 mmHg and
Daytime averaged BP < 135/85 mmHg and
Nighttime averaged BP ≥ 120/70 mmHg
Day-night masked uncontrolled hypertension (DN-MUCH)
Office BP < 140/90 mmHg and
Daytime averaged BP ≥ 135/85mmHg and
Nighttime averaged BP ≥ 120/70 mmHg
Isolated uncontrolled SBP
Daytime SBP ≥ 135 mmHg and/or Nighttime SBP ≥ 120 mmHg and
Daytime DBP < 85 mmHg and Nighttime DBP < 70 mmHg
Isolated uncontrolled DBP
Daytime DBP ≥ 85 mmHg and/or Nighttime DBP ≥ 70 mmHg and
Daytime SBP < 135 mmHg and Nighttime SBP < 120 mmHg
Uncontrolled SBP and DBP
Daytime SBP ≥ 135 mmHg and/or Nighttime SBP ≥ 120 mmHg and
Daytime DBP ≥ 85 mmHg and/or Nighttime DBP ≥ 70 mmHg

BP, blood pressure; SBP, systolic blood pressure; DBP, diastolic blood pressure.

**Table 2:** Demographic characteristics of patients with controlled office BP.

	CH	MUCH				*p*-Values (CH vs. MUCH)	*p*-Values *
		All	ID-MUCH	IN-MUCH	DN-MUCH		
*n* (%)	173 (31.8)	371	18 (3.3)	190 (34.9)	163 (30.0)		
Age, years	65.9 ± 8.7 ^a^	63.9 ± 9.1	59.1 ± 9.1 ^b^	66.5 ± 9.0 ^a^	61.5 ± 8.3 ^b^	0.018	<0.001
Sex							
Male, *n* (%)	52 (30.1) ^a^	217 (58.5)	12 (66.7) ^b^	103 (54.2) ^b^	102 (62.6) ^b^	<0.001	<0.001
Female, *n* (%)	121 (69.9)	154 (41.5)	6 (33.3)	87 (45.8)	61 (37.4)		
BMI, kg/m^2^	25.1 ± 2.9	25.2 ± 3.0	25.7 ± 3.3	25.1 ± 3.0	25.4 ± 2.9	0.450	0.441
Diabetes mellitus, *n* (%)	35 (20.2)	84 (22.6)	4 (22.2)	46 (24.2)	34 (20.9)	0.527	0.380
Smoking, *n* (%)	11 (6.4)	35 (9.4)	6 (33.3)	9 (4.7)	20 (12.3)	0.230	0.646
Drinking, *n* (%)	54 (31.2) ^a^	210 (56.6)	15 (83.3) ^b^	97 (51.1) ^b^	98 (60.1) ^b^	<0.001	<0.001
Amount of alcohol consumption						<0.001	<0.001
None (0 gr/day), *n* (%)	119 (69.2) ^a^	161 (43.8)	3 (17.6) ^b^	93 (49.5) ^b^	65 (39.9) ^b^		
Low, (0.2 to 6.4 gr/day), *n* (%)	33 (19.2) ^a^	95 (25.8)	4 (23.5) ^a^	51 (27.1) ^a^	40 (24.5) ^a^		
High (6.5 to 103.4 gr/day), *n* (%)	20 (11.6) ^a^	112 (30.4)	10 (58.8) ^b^	44 (23.4) ^c^	58 (35.6) ^b,c^		

* *p*-value given by the analysis of variance (continuous variables) or the Chi-square test (categorical variables), where the different superscript alphabets (a, b, and c) represent significant differences between groups as given by Tukey’s HSD or Games–Howell post hoc analysis at an alpha level of 0.05 (the analysis of variance) or the post hoc pairwise comparison at the Bonferroni =corrected alpha level of 0.0125 (the Chi-square test). CH, controlled hypertension; MUCH, masked uncontrolled hypertension; ID-MUCH, isolated daytime masked uncontrolled hypertension; IN-MUCH, isolated nighttime masked uncontrolled hypertension; DN-MUCH, daytime–nighttime masked uncontrolled hypertension; BMI, body mass index.

**Table 3:** Use of antihypertensive drugs in patients with controlled office BP.

	CH	MUCH				*p*-Values (CH vs. MUCH)	*p*-Values *
		All	ID-MUCH	IN-MUCH	DN-MUCH		
Number of antihypertensive drugs, *n*	1.7 ± 0.7	1.7 ± 0.7	1.8 ± 0.7	1.8 ± 0.8	1.7 ± 0.7	0.296	0.213
Antihypertensive drugs							
ACEi, *n* (%)	5 (2.9)	4 (1.1)	0 (0.0)	2 (1.1)	2 (1.2)	0.123	0.265
ARBs, *n* (%)	140 (80.9)	259 (69.8)	10 (55.6)	140 (73.7)	109 (66.9)	0.006	0.106
Beta blockers, *n* (%)	13 (7.5)	53 (14.3)	4 (22.2)	26 (13.7)	23 (14.1)	0.024	0.063
CCBs, *n* (%)	94 (54.3) ^a^	253 (68.2)	13 (72.2) ^a,b^	123 (64.7) ^a,b^	117 (71.8) ^b^	0.002	0.054
Diuretics, *n* (%)	37 (21.4)	75 (20.2)	5 (27.8)	43 (22.6)	27 (16.6)	0.753	0.801
Doses of antihypertensive drugs, standard dose	1.8 ± 0.9	2.0 ± 1.1	1.7 ± 0.9	2.0 ± 1.0	2.0 ± 1.2	0.118	0.129

* *p*-value given by the analysis of variance (continuous variables) or the Chi-square test (categorical variables), where the different superscript alphabets (a and b) represent significant differences between groups as given by Tukey’s HSD or Games–Howell post hoc analysis at an alpha level of 0.05 (the analysis of variance) or the post hoc pairwise comparison at the Bonferroni corrected alpha level of 0.0125 (the Chi-square test). CH, controlled hypertension; MUCH, masked uncontrolled hypertension; ID-MUCH, isolated daytime masked uncontrolled hypertension; IN-MUCH, isolated nighttime masked uncontrolled hypertension; DN-MUCH, daytime–nighttime masked uncontrolled hypertension; ACEi, angiotensin-converting enzyme inhibitors; ARBs, angiotensin receptor blockers; CCBs, calcium channel blockers.

**Table 4:** Clinical characteristics of patients with controlled office BP.

	CH	MUCH				*p*-Values (CH vs. MUCH)	*p*-Values *
		All	ID-MUCH	IN-MUCH	DN-MUCH		
Cardiovascular disease, *n* (%)	27 (15.6)	58 (15.6)	3 (16.7)	26 (13.7)	29 (17.8)	0.994	0.656
Chronic kidney disease, *n* (%)	6 (3.5)	12 (3.2)	0 (0.0)	10 (5.3)	2 (1.2)	0.887	0.164
eGFR, ml/min/1.73 m^2^	84.9 ± 13.5 ^a^	88.1 ± 13.9	93.8 ± 9.5 ^a^	85.8 ± 14.6 ^a^	90.1 ± 13.0 ^b^	0.013	<0.001
UACR	14.4 ± 36.3	27.6 ± 116.7	9.8 ± 8.5	22.0 ± 108.0	36.2 ± 131.7	0.048	0.909
HbA1C, %	5.9 ± 0.7	5.8 ± 0.6	5.8 ± 0.6	5.8 ± 0.6	5.8 ± 0.6	0.384	0.608
Office SBP, mmHg	121.5 ± 8.4 ^a^	126.2 ± 7.3	126.1 ± 8.1 ^a,b,c^	124.4 ± 7.7 ^b^	128.4 ± 6.2 ^c^	<0.001	0.001
Office DBP. mmHg	72.1 ± 6.0 ^a^	78.4 ± 6.0	80.5 ± 6.5 ^b,c^	75.9 ± 5.7 ^b^	81.0 ± 5.1 ^c^	<0.001	<0.001
24 h SBP, mmHg	114.0 ± 6.4 ^a^	124.4 ± 7.8	121.9 ± 4.7 ^b^	121.1 ± 6.4 ^b^	128.6 ± 7.5 ^c^	<0.001	<0.001
24 h DBP, mmHg	70.3 ± 4.8 ^a^	81.0 ± 5.9	77.9 ± 5.1 ^b^	77.6 ± 4.5 ^b^	85.3 ± 4.6 ^c^	<0.001	<0.001
Daytime SBP, mmHg	118.3 ± 7.6 ^a^	126.9 ± 9.0	131.6 ± 6.8 ^c^	121.6 ± 6.5 ^b^	132.5 ± 7.9 ^c^	<0.001	<0.001
Daytime DBP, mmHg	73.5 ± 5.7 ^a^	83.1 ± 7.3	85.6 ± 5.1 ^c^	78.2 ± 5.2 ^b^	88.6 ± 5.1 ^c^	<0.001	<0.001
Nighttime SBP, mmHg	105.4 ± 6 ^a^	119.6 ± 10.1	104.2 ± 5.2 ^a^	119.7 ± 9.2 ^b^	121.2 ± 10.2 ^b^	<0.001	<0.001
Nighttime DBP, mmHg	63.7 ± 4.3 ^a^	76.6 ± 6.8	63.6 ± 4.4 ^a^	75.9 ± 5.7 ^b^	78.9 ± 6.4 ^b^	<0.001	<0.001

* *p*-value given by the analysis of variance (continuous variables) or the Chi-square test (categorical variables), where the different superscript alphabets (a, b, and c) represent significant differences between groups as given by Tukey’s HSD or Games–Howell post hoc analysis at an alpha level of 0.05 (the analysis of variance) or the post hoc pairwise comparison at the Bonferroni corrected alpha level of 0.0125 (the Chi-square test). CH, controlled hypertension; MUCH, masked uncontrolled hypertension; ID-MUCH, isolated daytime masked uncontrolled hypertension; IN-MUCH, isolated nighttime masked uncontrolled hypertension; DN-MUCH, daytime–nighttime masked uncontrolled hypertension; eGFR, estimated glomerular filtration rate; UACR, spot urine albumin creatinine ratio; SBP, systolic blood pressure; DBP, diastolic blood pressure.

**Table 5:** Comparison between patients with isolated uncontrolled DBP and uncontrolled SBP and DBP in patients with isolated nighttime masked uncontrolled hypertension.

	Patients with Isolated Uncontrolled DBP	Patients with Uncontrolled SBP and DBP	*p*-Values	*p*-Values *
*n*	98	78		
Age	64.9 ± 8.0	67.7 ± 9.9	0.043	
Gender, *n* (%)				
Male	61 (62.2)	36 (46.2)	0.033	
Female	37 (37.8)	42 (53.8)		
BMI, kg/m^2^	25.2 ± 3.0	25.1 ± 3.1	0.791	
Smoking, *n* (%)	8 (8.2)	1 (1.3)	0.040	
Drinking, *n* (%)	59(60.2)	32 (41.0)	0.011	
Amount of alcohol consumption			0.032	
None (0 gr/day), *n* (%)	39 (40.2)	46 (59.7)		
Low (0.2 to 6.4 gr/day), *n* (%)	30 (30.9)	18 (23.4)		
High (6.5 to 103.4 gr/day), *n* (%)	28 (28.9)	13 (16.9)		
Cardiovascular disease, *n* (%)	8 (8.2)	13 (16.7)	0.084	
Diabetes mellitus, *n* (%)	21 (21.4)	19 (24.4)	0.645	
Office BP categories, *n* (%)				
Optimal	35 (35.7)	17 (21.8)	0.073	
Normal	45 (45.9)	38 (48.7)		
High normal	18 (18.4)	23 (29.5)		
Chronic kidney disease				
Stage 1	46 (46.9)	34 (43.6)	0.802	
Stage 2	47 (48.0)	41 (52.6)		
Stage 3	5 (5.1)	3 (3.8)		
SD of 24 h SBP	12.4 ± 3.3	14.3 ± 3.5	<0.001	0.003
SD of 24 h DBP	9.7 ± 2.0	10.1 ± 2.1	0.203	0.165

* Multivariate analysis adjusted for age and sex. BMI, body mass index; BP, blood pressure; SD, standard deviation; SBP, systolic blood pressure; DBP, diastolic blood pressure.

## Data Availability

The datasets generated and/or analyzed during the current study are available from the corresponding author upon reasonable request.

## Abbreviations

ACEi	Angiotensin-converting enzyme inhibitors
ARBs	Angiotensin receptor blockers
BMI	Body mass index
BP	Blood pressure
CCBs	Calcium channel blockers
CH	Controlled hypertension
CKD-EPI	Chronic Kidney Disease-Epidemiology Collaboration
CVD	Cardiovascular disease
DBP	Diastolic blood pressure
DN-MUCH	Daytime and nighttime masked uncontrolled hypertension
eGFR	Estimated glomerular filtration rate
IDACO	International Database on Ambulatory Blood Pressure in Relation to Cardiovascular Outcomes
ID-MUCH	Isolated daytime masked uncontrolled hypertension
INH	Isolated nighttime hypertension
IN-MUCH	Isolated nighttime masked uncontrolled hypertension
MUCH	Masked uncontrolled hypertension
NYHA	New York Heart Association
SBP	Systolic blood pressure
SD	Standard deviation
UACR	Spot urine albumin creatinine ratio
